# Co-Regulation of Very Fast Chilling Treatment and the Follow-Up Storage Temperature on Meat Tenderness Through Glycolysis

**DOI:** 10.3390/foods14172932

**Published:** 2025-08-22

**Authors:** Yuqiang Bai, Chi Ren, Saisai Wu, Chengli Hou, Xin Li, Dequan Zhang

**Affiliations:** Institute of Food Science and Technology, Chinese Academy of Agricultural Sciences/Key Laboratory of Agro-Products Quality & Safety in Harvest, Storage, Transportation, Management and Control, Ministry of Agriculture and Rural Affairs, Beijing 100193, China; yuqiangbai_1844@163.com (Y.B.); candymce@163.com (C.R.); wssaxiu1736@163.com (S.W.); houchengli@163.com (C.H.); dequan_zhang0118@126.com (D.Z.)

**Keywords:** tenderness, glycolytic enzymes, activity, phosphorylation, acetylation

## Abstract

The effects of storage temperature (4 °C, −1 °C, and −4 °C) after the very fast chilling (VFC) treatment on the glycolysis in lamb were investigated. The meat tenderness, glycolytic rates, activity, phosphorylation, and acetylation levels of glycolytic enzymes in meat stored at different temperatures were measured. It was shown that there was no significant difference in the degradation degree of desmin and troponin T in meat at different storage temperatures after VFC treatment (*p* < 0.05). The decrease rate of pH and ATP in meat was the same under different storage temperatures. The promoted phosphorylation and acetylation levels of phosphofructokinase (PFKM) and phosphoglycerate kinase (PGK) and inhibited acetylation level of aldolase (ALDOA) in the samples stored at different temperatures maintained the same glycolytic rate. In conclusion, chilling treatment is the key step in improving meat tenderness rather than storage temperature, which is achieved by the increased phosphorylation of ALDOA, PFKM, and PGK and decreased acetylation of ALDOA. It indicated that the chilling rate promoted the improvement of meat quality mainly by delaying glycolysis compared to the storage temperature.

## 1. Introduction

Pre-rigor fresh meat is widely recognized in China with a consumption proportion over 50%, due to its delicious taste [1,2]. Since there is no aging process in the production of pre-rigor meat, the production space is saved, the energy consumption and storage losses are reduced [3]. The texture, water holding capacity (WHC), and flavor of meat vary at different postmortem stages [4]. Pre-rigor meat has lower free water content, better WHC and color, and a richer taste compared with post-rigor meat [2]. The roast loss of pre-rigor mutton is lower, and the meat quality score was significantly higher than that in samples at the rigor stage [1]. Therefore, the technology to maintain the pre-rigor meat quality is particularly important, which remains to be further explored.

Very fast chilling (VFC) refers to the process by which the core temperature of a carcass declines rapidly to −1 °C within 5 h after slaughter (chilling rate ≥ 7.6 °C/h) [5]. Previous studies have shown that the VFC treatment is an efficient and beneficial way of carcass chilling [6], which maintains higher meat quality. Cold shortening can be inhibited by rapid chilling to reach a core temperature of –1 °C within 5 h [7]. Compared with 4 °C storage, the VFC treatment significantly inhibited the total bacterial count on the carcass surface and extended the shelf life [8]. The VFC treatment delayed the degradation of flavor nucleotides and volatile flavor compounds, maintaining the flavor of fresh meat [9]. It has been proved that the VFC treatment affected rigor and improved meat tenderness [10]. The postmortem physiological and biochemical processes, including glycolysis, were also delayed in meat with improved tenderness after the VFC treatment [11]. It was found that the VFC treatment delayed glycolysis by promoting the phosphorylation levels of aldolase (ALDOA), lactate dehydrogenase (LDH), and triosephosphate isomerase (TPI1) and inhibiting their acetylation levels, consequently improving meat quality [12].

Storage temperature is also an important environmental factor affecting meat quality. Previous studies have shown that there were differences in meat color, degree of protein denaturation, muscle microstructure, and water distribution of meat stored at different temperatures [13,14,15]. Both the chilling rates and storage temperatures affect the glycolytic rate in meat through post-translational modifications of glycolytic enzymes [16]. Compared with meat stored at 25, 15, and 4 °C, phosphorylation levels of sarcoplasmic protein and the decline rate of pH in meat stored at −1.5 °C were significantly inhibited [16]. The cold environments decreased the total protein acetylation level in tadpole livers, and the content of acetyl coenzyme A was higher. It indicated that the expression of acetylated proteins may be regulated by factors other than genes of histone acetyltransferase or histone deacetylase [17]. The colony composition varied in meat stored at 4, −1, −4, and −9 °C after the VFC treatment with a chilling rate of 14.52 °C/h [8,18]. Storing at −1.5, −4, and −9 °C after the VFC treatment showed positive effects on extending shelf life; the maximums were 14, 28, and 79 d, respectively [18]. Previous studies have shown that both the chilling rates and storage temperatures changed meat quality by influencing glycolysis in meat. However, it is still unclear how different storage temperatures affect meat quality after the same chilling rate. Therefore, the tenderness and glycolysis changes in meat samples stored at different temperatures after VFC were explored in this study. It was helpful to develop more efficient and energy-saving VFC technologies.

## 2. Materials and Methods

### 2.1. Experimental Design

The longissimus thoracis muscles were purchased from Beijing Ershang Meat Food Group Co., Ltd., Beijing, China, according to the guidelines of the Animal Care and Ethics Committee for animal experiments at the Institute of Food Science and Technology, Chinese Academy of Agricultural Sciences. These samples were used to explore the effects of different storage temperatures after VFC treatment on meat quality and glycolysis. These samples were randomly selected from the same batch of Small Tail Han sheep (7 months old, carcass weight was 24.2 ± 1.6 kg) with the same animal background and slaughter process at a local slaughterhouse. The left side of the longissimus thoracis muscle (around 650 g, 25 cm × 8 cm × 3 cm) was collected from thirty male Small Tail Han sheep carcasses within 1 h postmortem. The muscles were completely wrapped by plastic film and kept at −35 °C (air velocity of 3 m/s) for chilling. The duration of the chilling treatment was 1.5 h. Following VFC treatment, the core temperature of the meat reached −1 °C. The very fast chilled muscles were divided into three groups randomly (*n* = 10): (1) chilling group (Chilling), stored at 4 °C; (2) supercooling group (Supercooling, serving as the Control group), stored at −1 °C; (3) superchilling group (Superchilling), stored at −4 °C. The meat was stored in incubators (MIR-154-PC, Panasonic, Osaka, Japan). The core temperature of meat was monitored by a multi-channel temperature monitoring instrument (LK-XU, Blue Bright, Changzhou, China) continuously. Samples were obtained at 0 h, 2 h, 12 h, 1 d, 3 d, and 5 d throughout the storage period. The Supercooling group (−1 °C) was used as the control to analyze the effects of Chilling (4 °C) and Superchilling (−4 °C) on meat quality and glycolysis.

### 2.2. pH

The pH meter (Testo205 pH meter, Lenzkirch, Germany) with temperature compensation was calibrated by calibration buffer with pH 4.0, 7.0, and 10.0. The probe of the meter was inserted into the meat with a depth of 2 cm. The pH of the sample was measured using the automatic hold function (AUTO HOLD) until the reading fixed and the AUTO HOLD light came on. All measurement was completed within 120 s.

### 2.3. Shear Force

Approximately 65 g of meat was kept at 71 °C for 35 min, cooled by running water for 30 min, and then cut into 6 strips (1.0 cm × 1.0 cm × 1.5 cm) along the direction of muscle fibers [19]. The shear force was measured by a texture analyzer (TA-XT plus, Stable Micro System, London, UK) with an HDP/BSW probe. The moving speed of the probe is 2.0 mm/s before shearing, 1.0 mm/s during shearing, and 10.0 mm/s after shearing [4].

### 2.4. Degradation Degree of Desmin and Troponin T

The SDS-PAGE electrophoresis was carried out at 150 V, and then the PVDF membrane (polyvinylidene fluoride, Millipore, Billerica, MA, USA) was used for Western blotting at 100 V for 180 min [16]. The membrane was incubated with antibodies against desmin (A0699, Abclonal, Wuhan, China) and troponin T (A1157, Abclonal, Wuhan, China).

### 2.5. Contents of Glycolytic Metabolites

According to the guidelines of glycogen, lactate, and ATP kits (Solarbio, Beijing, China), 0.1 g of meat mixed with the extraction solution provided by the kits was then homogenized in an ice bath with a homogenizer (T25, IKA, Staufen, Germany), centrifuged at 9500 rpm at 4 °C for 5 min, and incubated at room temperature for 10 min. The absorbance values were detected at 620, 570, and 340 nm using a microplate reader (Spark, Tecan, Austria), respectively. The measurement was repeated three times, and the average value was taken. The units of glycogen, lactate, and ATP were mg/g, mmol/g and μmol/g, respectively.

### 2.6. Protein Phosphorylation and Acetylation Levels

The phosphorylation and acetylation levels of glycolytic enzymes (ALDOA, PFKM, PGK, PYGM, TPI1, and LDH) were determined by SDS-PAGE electrophoresis (Bio-Rad, Hercules, CA, USA) and Western blotting [12]. The SDS-PAGE electrophoresis was performed using 10% separation gel at 100 V for 90 min. A PVDF membrane was used for determining phosphorylation levels, and the membrane was transferred for 150 min under 100 V. When measuring acetylation levels, a nitrocellulose membrane (NC membrane) was used and was transferred for 200 min under 100 V.

### 2.7. Activity of Glycolytic Enzymes

Referencing the instructions of kits (Solarbio, Beijing, China), the mixture in a ratio of mass (g) to volume of the extracted liquid (mL) of 1:10 was homogenized. After centrifugation, the supernatant was incubated with buffer, and the absorbance value was measured at 340 nm by a microplate reader (Spark, Tecan, Männedorf, Switzerland). The measurement was repeated three times. The unit of enzyme activity was U/g.

### 2.8. Statistical Analysis

The SPSS Statistic 21.0 (IBM Corporation, Armonk, NY, USA) and general linear model (GLM) were used to analyze the data. The treatment, storage time, and their interaction were considered as fixed effects, and the carcass as a random effect. The significance of data was evaluated by Fisher’s Protected Least Significant Difference (LSD) test (*p* < 0.05). All experiments were conducted in triplicate, and the data were presented as mean and standard error.

## 3. Results

### 3.1. The Tenderness of Meat at Different Storage Temperature

**Figure 1:**
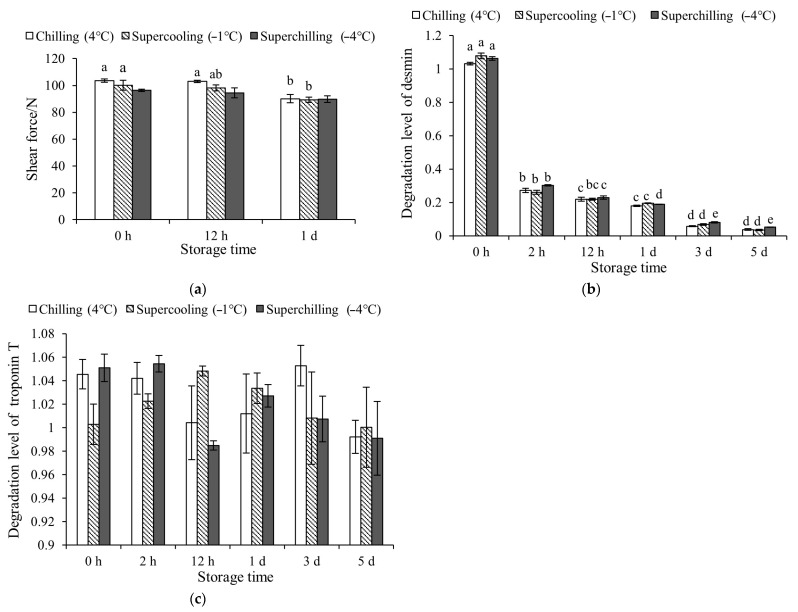
Effect of different storage temperature on the shear force degradation level of desmin and troponin T in lamb at 0 h, 2 h, 12 h, 1 d, 3 d, and 5 d during storage. (**a**) Shear force under different storage temperatures. (**b**) Desmin degradation levels in lamb under different storage conditions; (**c**) Troponin T degradation levels in lamb under different storage conditions. a–e: letters indicate significant intra-group differences over different storage durations (*p* < 0.05).

The shear force of meat samples in three groups was the same during storage. After VFC, the shear force of meat in Chilling (4 °C) and Supercooling (−1 °C) groups decreased at 1 d of storage (Figure 1). It meant that the storage temperature did not change the meat tenderness after VFC treatment. The degradation degree of desmin in three groups decreased and then stabilized during 3–5 d of storage. There was no significant difference in the degradation degree of desmin and troponin T in meat (*p* > 0.05) among three groups during storage.

### 3.2. The Glycolytic Rates of Meat at Different Storage Temperature

The pH of meat in the Supercooling (−1 °C) group was significantly higher than that in the Chilling (4 °C) group during 2–12 h of storage (*p* < 0.05, Table 1). From 12 h to 1 day, the Superchilling group (−4 °C) exhibited a significantly higher pH compared to the Supercooling (−1 °C) group. No significant differences in pH were observed among the three groups at 0 h, 3 d, and 5 d of storage. During storage, the pH of meat in the three groups decreased. The pH in the Chilling (4 °C) and Supercooling (−1 °C) groups decreased to the lowest at 1 d of storage, while that in the Superchilling (−4 °C) group decreased on 3 d of storage.

The ATP content in Supercooling (−1 °C) and Superchilling (−4 °C) groups was not significantly different from that in the Chilling group (4 °C) at 12 h of storage (*p* > 0.05). The ATP content in the Superchilling (−4 °C) group was higher than that in the Supercooling (−1 °C) group. The ATP content in the Superchilling (−4 °C) group was highest among the three groups during 1 d to 3 d of storage. The ATP content in the three groups decreased gradually during storage. The ATP content in the Chilling (4 °C) and Supercooling (−1 °C) groups tended to be stable after 3 d of storage.

Throughout the storage period, the Supercooling group (−1 °C) exhibited the highest glycogen content, whereas the Superchilling group (−4 °C) showed the lowest levels. The content of glycogen in three groups decreased gradually during storage after VFC treatment.

Between 12 h and 3 d of storage, the Superchilling group (−4 °C) exhibited the highest lactate content. The lactate content did not differ significantly in the Chilling (4 °C) and Supercooling (−1 °C) groups from 1 to 3 d of storage (*p* > 0.05). The lactate content in the Chilling (4 °C) and Supercooling (−1 °C) groups increased gradually during storage.

### 3.3. Protein Phosphorylation Levels at Different Storage Temperature

**Figure 2:**
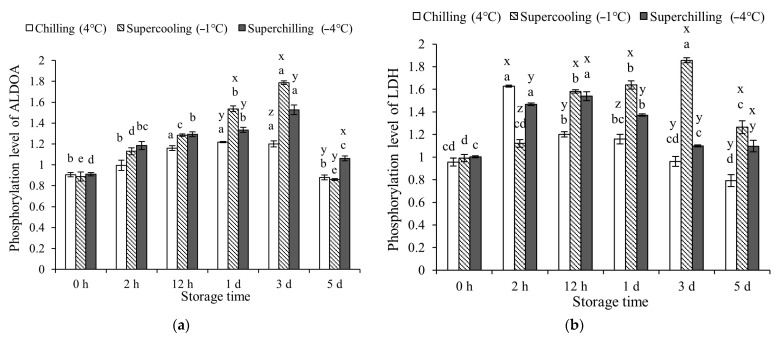

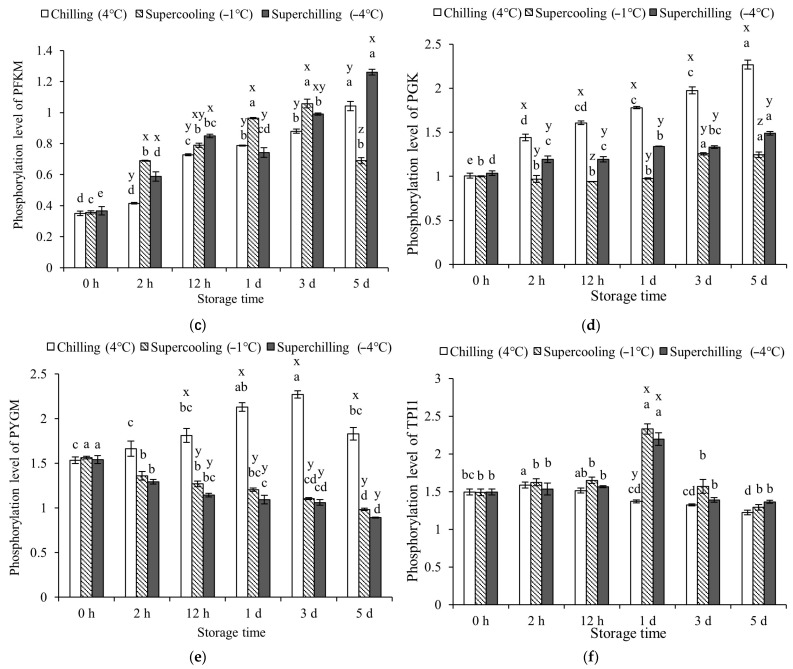
Phosphorylation levels of glycolytic enzymes in lamb at 0 h, 2 h, 12 h, 1 d, 3 d, and 5 d during storage at different storage temperatures. (**a**) Fructose–bisphosphate aldolase A, ALDOA. (**b**) L-lactate dehydrogenase, LDH. (**c**) Phosphofructokinase, PFKM. (**d**) Phosphoglycerate kinase, PGK. (**e**) Glycogen phosphorylase, PYGM. (**f**) Triosephosphate isomerase 1, TPI1. x–z: letters denote statistically significant inter-group differences at the same time point (*p* < 0.05). a–e: letters indicate significant intra-group differences over different storage durations (*p* < 0.05).

From 1 d to 3 d of storage, Supercooling (−1 °C) exhibited a stronger promotive effect on ALDOA phosphorylation compared to the other groups (*p* < 0.05, Figure 2). Within the first 12 h of storage, the three temperature treatments had a comparable effect on the phosphorylation level of ALDOA. At the later stages of storage, LDH phosphorylation significantly declined in both the Chilling (4 °C) and Superchilling (−4 °C) groups, with levels notably lower than those in the Supercooling group (−1 °C). The phosphorylation level of PFKM in the Chilling group (4 °C) was reduced compared to that in the Supercooling group (−1 °C) at 2 h, 1 d, and 3 d. No significant differences in PFKM phosphorylation were found between the Supercooling (−1 °C) and Superchilling (−4 °C) groups at 2 h, 12 h, and 3 d. Throughout the storage period, PGK phosphorylation was significantly enhanced under Chilling (4 °C) conditions. Moreover, the effect of Superchilling (−4 °C) on PGK phosphorylation was comparable to that of Supercooling (−1 °C). Chilling (4 °C) storage significantly promoted the phosphorylation level of PYGM during 12 h-5 d of storage. The effect of Supercooling (−1 °C) and Superchilling (−4 °C) on the phosphorylation of PYGM were the same during storage. The phosphorylation level of TPI1 in Superchilling (−4 °C) and Supercooling (−1 °C) groups was the same during storage, which was higher than that in the Chilling (4 °C) group at 1 d. The phosphorylation levels of ALDOA, LDH, and TPI1 in the three groups increased and then decreased during storage. The phosphorylation levels of PFKM in the Chilling (4 °C) and Superchilling (−4 °C) groups remained increased. The PGK phosphorylation levels in the three groups increased, while the phosphorylation level of PYGM in the Superchilling (−4 °C) and Supercooling (−1 °C) groups decreased.

### 3.4. Protein Acetylation Levels at Different Storage Temperature

**Figure 3:**
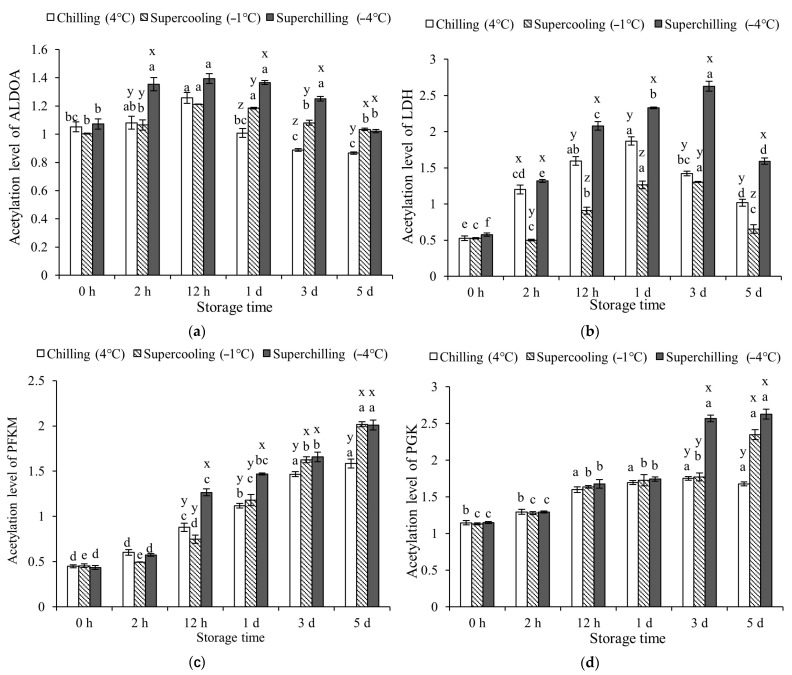

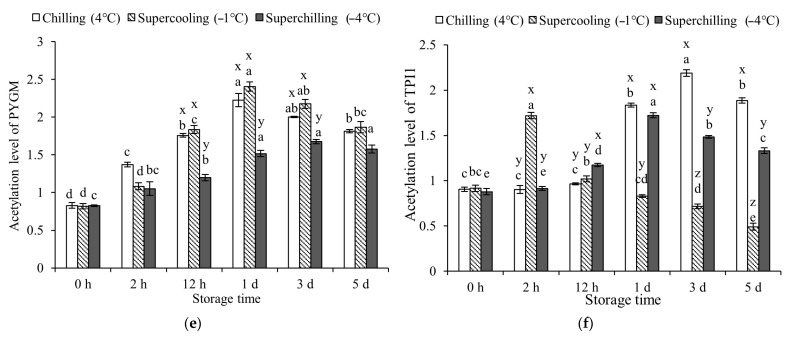
Acetylation levels of glycolytic enzymes in lamb at 0 h, 2 h, 12 h, 1 d, 3 d and 5 d during storage at different storage temperatures. (**a**) Fructose–bisphosphate aldolase A, ALDOA. (**b**) L-lactate dehydrogenase, LDH. (**c**) Phosphofructokinase, PFKM. (**d**) Phosphoglycerate kinase, PGK. (**e**) Glycogen phosphorylase, PYGM. (**f**) Triosephosphate isomerase 1, TPI1. x–z: letters denote statistically significant inter-group differences at the same time point (*p* < 0.05). a–f: letters indicate significant intra-group differences over different storage durations (*p* < 0.05).

There was no significance in the acetylation level of ALDOA in the Chilling (4 °C) and Supercooling (−1 °C) groups within 12 h (*p* > 0.05, Figure 3). The acetylation level of ALDOA was significantly promoted by Superchilling (−4 °C) storage (*p* < 0.05). Compared with Supercooling (−1 °C) storage, Superchilling (−4 °C) storage significantly promoted the acetylation level of LDH. Chilling (4 °C) storage significantly promoted the acetylation level of LDH at 12 h, 1 d, and 5 d of storage. When stored for 12 h and 1 d, the acetylation level of PFKM in the Chilling group (4 °C) was consistent with that in the Supercooling group (−1 °C). Subsequently, the acetylation level of PFKM in the Supercooling (−1 °C) and Superchilling (−4 °C) groups was consistent and higher than that in the Chilling group (4 °C). Three storage temperatures did not change the acetylation level of PGK within 1 d of storage. Superchilling (−4 °C) storage inhibited the acetylation level of PYGM during 12 h to 3 d of storage. The effect of Chilling (4 °C) and Supercooling (−1 °C) storage on the acetylation level of PYGM was the same during storage. At 2 h of storage, the Supercooling (−1 °C) group exhibited the highest acetylation level of TPI1. During the storage period, the acetylation levels of ALDOA, LDH, PYGM, and TPI1 showed an initial increase followed by a decline, whereas those of PFKM and PGK continuously increased over time.

### 3.5. Activity of Glycolytic Enzymes at Different Storage Temperatures

**Figure 4:**
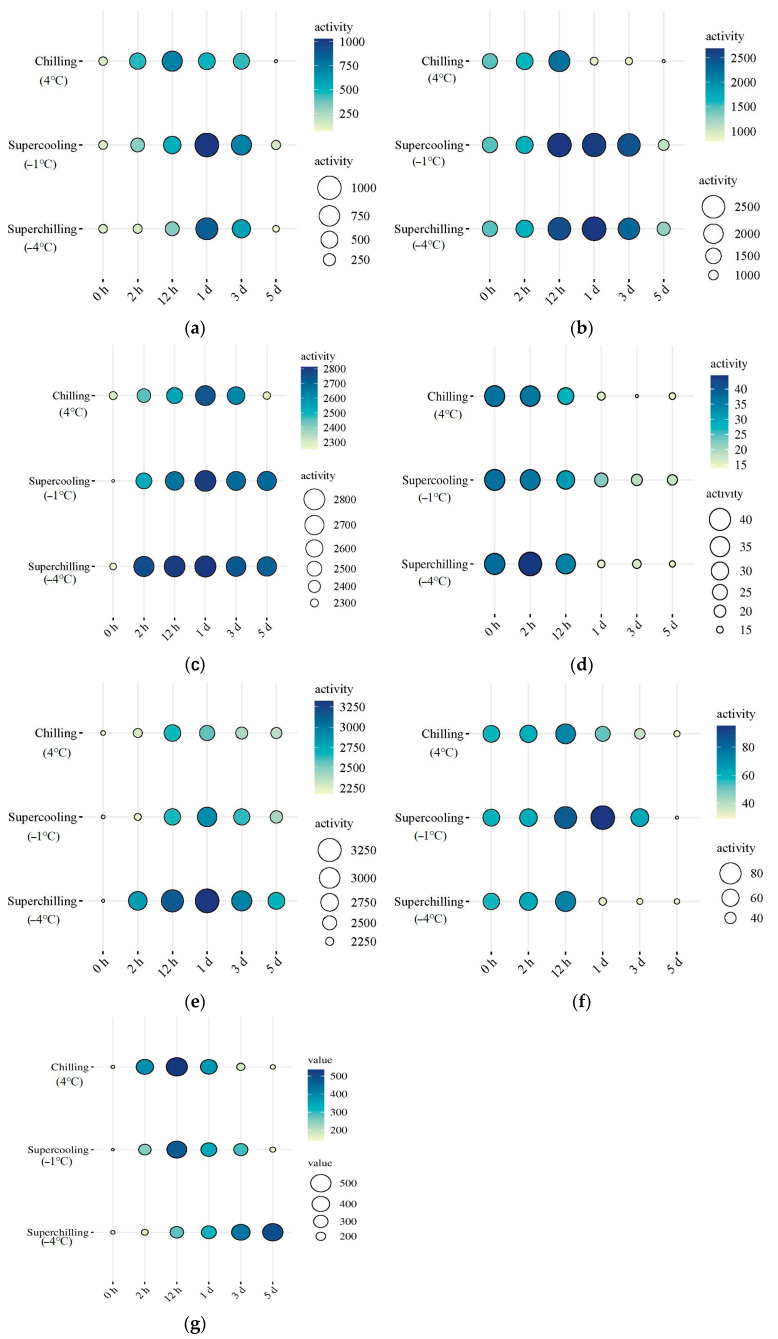
Activity of the glycolytic enzymes in lamb at 0 h, 2 h, 12 h, 1 d, 3 d and 5 d during storage at different storage temperatures. (**a**) Fructose–bisphosphate aldolase A, ALDOA. (**b**) L-lactate dehydrogenase, LDH. (**c**) Phosphofructokinase, PFKM. (**d**) Phosphoglycerate kinase, PGK. (**e**) Glycogen phosphorylase, PYGM. (**f**) Triosephosphate isomerase 1, TPI1. (**g**) Pyruvate kinase, PKM.

The Supercooling (−1 °C) group exhibited the highest activities of ALDOA, PFKM, and TPI1 at 1 d of storage, while LDH activity peaked in this group at 12 h (Figure 4). From 1 d to 5 d, ALDOA activity in the Superchilling (−4 °C) group was significantly higher than that in the Chilling (4 °C) group (*p* < 0.05). At 12 h, PGK activity in the Superchilling (−4 °C) group was also greater than that in the Chilling (4 °C) group. Additionally, PGK activity in the Supercooling (−1 °C) group was significantly higher than in both the Chilling (4 °C) and Superchilling (−4 °C) groups at 1 d. However, LDH activity in the Supercooling (−1 °C) group was the lowest between 12 h and 5 d. No significant difference in LDH activity was observed between the Chilling (4 °C) and Superchilling (−4 °C) groups from 12 h to 1 d (*p* > 0.05). The activity of PFKM in Supercooling (−1 °C) group was lower than that in Chilling (4 °C) and Superchilling (−4 °C) groups during storage. The activity of PFKM in Chilling group (4 °C) was lower than that in the Superchilling (−4 °C) group during 2–12 h of storage. The activity of six glycolytic enzymes in three groups increased and then decreased during storage. The activity of pyruvate kinase (PKM) in Supercooling (−1 °C) group was higher than that in the Superchilling (−4 °C) group during 2 h-1 d of storage. The activity of PKM in Supercooling (−1 °C) and Chilling (4 °C) groups increased first and then decreased, which increased during storage in Superchilling (−4 °C) group.

## 4. Discussion

### 4.1. Effect of Storage Temperature on Meat Tenderness

Previous studies have shown that compared to conventional chilling (without VFC treatment), the VFC treatment significantly delayed glycolysis and improved meat tenderness [10,12]. It has been proved that the VFC treatment improved meat tenderness by activating calpain and promoting the degradation of skeleton proteins [10]. This study was carried out and focused on the effects of different storage temperatures after VFC on meat quality and its metabolism as a follow-up exploration. Compared with the control group of −1 °C storage, the improvement of VFC treatment on meat tenderness was not changed by different storage temperatures. Meat tenderness is directly related to the degradation of myofibrillar protein during aging [20]. The desmin can be used as a biomarker for postmortem protein degradation. The degradation of troponin-T is a marker of meat tenderization and aging postmortem. Desmin is located on the muscle fiber membrane and nuclear membrane, playing a role in connecting and fixing myofibrils and maintaining the normal function of muscle fiber [21]. Troponin T is a myofilament protein that mainly binds to tropomyosin, which is directly involved in muscle contraction controlled by Ca^2+^ [22]. Degradation of troponin T disrupts the integrity of myofilaments, alters the interaction between actin and myosin, and leads to fragmentation of myofibrils [23,24]. It was proved that VFC increased the content of Ca^2+^ and activated μ-calpain at early postmortem, leading to degradation of skeleton proteins and improvement of meat tenderness [10]. The degradation level of skeletal proteins confirmed the inhibitory effect of VFC on rigor. The consistent degree of oxidation and changes in total bacterial count of meat stored at −4 and −9 °C indicated that the effect of −4 °C and lower-temperature storage on meat quality may be the same after VFC treatment [18]. In terms of tenderness, the effect of storage temperatures on tenderness after VFC was consistent. Different storage temperatures after VFC treatment did not affect the tenderness of meat during storage. Combined with the existing results, VFC significantly improved meat tenderness; it means that VFC (chilling rates) vitals are better in the improvement of tenderness than storage temperatures.

### 4.2. Effect of Storage Temperature on Glycolysis

After confirming the consistent improvement effect of different storage temperatures on tenderness after VFC treatment, the changes in glycolysis at different storage temperatures were further studied to better explain the mechanism by which VFC improved tenderness. Compared with Supercooling (−1 °C) storage, the same decline rate of pH and ATP in meat under Chilling (4 °C) storage indicated the same effect on glycolysis. The decrease in pH is due to the accumulation of lactate and H+ during glycolysis and ATP hydrolysis [12,25]. In terms of the decline rate of pH and ATP, the delayed effect of Superchilling (−4 °C) storage was better than the Supercooling (−1 °C) and Chilling (4 °C) storage. The storage temperature after VFC only changed the decline rates of pH and ATP but did not change the degree of decline. The glycogen content in meat stored at Superchilling (−4 °C) decreased, and the lactate content increased significantly. In terms of the changes in glycogen and lactate content, Superchilling (−4 °C) storage after VFC promoted the degradation of glycogen and accumulation of lactate, which may be due to the destruction of muscle structure, manifested as frostbite of muscle fibers [26]. The high content of IMP may lead to the separation of myosin from actin, thus weakening the actin–myosin bridge [27]. In general, Supercooling (−1 °C) and Superchilling (−4 °C) storage temperatures did not change the effect of VFC treatment on the delayed glycolysis and improved meat tenderness, indicating that the VFC treatment is the key step to improve meat tenderness.

### 4.3. Effect of Storage Temperature on Glycolytic Enzymes

The glycolytic rate was closely regulated by the activity of glycolytic enzymes, which in turn was affected by protein post-translational modifications (PTMs), such as phosphorylation and acetylation, as well as by storage temperature [12,16]. Previous studies have demonstrated that chilling conditions can enhance both phosphorylation and acetylation levels of glycolytic enzymes, thereby contributing to a deceleration of the glycolytic process [12]. In this study, Chilling (4 °C) storage inhibited the acetylation level of TPI1 while promoting the phosphorylation of PYGM and both the acetylation and phosphorylation of PGK. The phosphorylation level of ALDOA acetylation and phosphorylation levels of TPI1 were not significantly different between Chilling (4 °C) and Supercooling (−1 °C) groups. The Superchilling (−4 °C) storage further promoted the phosphorylation level of PGK and TPI1, as well as the acetylation level of PFKM and PGK. These findings suggested that the observed changes in the PTMs of PFKM, PGK, PYGM, TPI1, and ALDOA in the Supercooling (−1 °C) and Superchilling (−4 °C) groups were consistent with those previously reported in VFC-treated samples with the chilling rate of 25.1 °C/h. It meant that the increased phosphorylation levels of PYGM, PGK, and TPI1, increased acetylation levels of PFKM and PGK, and decreased acetylation level of TPI1 were the main regulatory factors in delaying glycolysis after VFC treatment. The VFC treatment with a chilling rate of 25.1 °C/h effectively suppresses glycolysis through PTMs of glycolytic enzymes [12]. In this study, the higher activity of ALDOA and PFKM delayed the glycolysis in the meat stored in the Superchilling (−4 °C) storage. The same activity of PGK in three groups during 2 h of storage indicated the key role of the VFC treatment on the PGK activity. The activity changes in PYGM and LDH in the meat stored at Superchilling (−4 °C) conditions promoted the degradation of glycogen and the accumulation of lactate. The increased activity of PYGM was due to the decreased acetylation level, which reduced its inhibitory effect on PYGM activity [28]. The increased activity of LDH was due to its high phosphorylation at early storage [29]. The lower activity of PKM in the Superchilling (−4 °C) group indicated the lower rate of pyruvate production and delayed glycolysis. From the perspectives of the enzyme activity, ALDOA, PFKM, and PGK were the key enzymes in regulating glycolysis in meat after the VFC treatment. Combined with the results of activity and phosphorylation and acetylation levels of glycolytic enzymes, ALDOA, PFKM, and PGK played a key role in regulating glycolysis in the VFC treatment.

The activity changes in PGK in the Chilling (4 °C) and Supercooling (−1 °C) groups were basically consistent with the phosphorylation and acetylation levels. The increased phosphorylation and acetylation levels of PGK contribute to increased ATP and NADPH production during glycolysis [30,31]. The VFC treatment has been shown to enhance PGK phosphorylation, which helps slow glycolysis [12]. The decreased acetylation of ALDOA was associated with enhanced activity, while increased phosphorylation of ALDOA was linked to glycolytic suppression [25,32]. For PFKM, both phosphorylation and acetylation were found to positively regulate its activity, further contributing to delayed glycolysis [33,34]. The higher activity of glycolytic enzymes after VFC treatment may not only regulate glycolysis directly but may also participate in other metabolic processes, such as protein kinase activity of pyruvate kinase, in addition to traditional kinase functions in glycolysis [35,36].

## 5. Conclusions

The results of this study confirmed that meat tenderization and glycolysis were not affected by different storage temperatures: Chilling (4 °C), Supercooling (−1 °C), and Superchilling (−4 °C) after VFC treatment. These storage conditions maintained delayed glycolysis by increasing phosphorylation levels of ALDOA, PFKM, and PGK, and decreasing the acetylation level of ALDOA. In general, the storage temperatures after VFC treatment at −35 °C in this study had the same improvement in meat quality, providing a theoretical basis for the development of VFC treatment protocols and optimization of storage temperature.

## Figures and Tables

**Table 1:** The glycolytic rates in lamb with different storage temperatures after VFC.

		0 h	2 h	12 h	1 d	3 d	5 d
pH	Chilling (4 °C)	6.05 ± 0.24 ^a^	5.79 ± 0.1 5^ya^	5.20 ± 0.03 ^zb^	5.14 ± 0.02 ^yc^	5.10 ± 0.05 ^c^	5.20 ± 0.09 ^c^
	Supercooling (−1 °C)	6.18 ± 0.13 ^a^	6.08 ± 0.18 ^xa^	5.34 ± 0.05 ^yb^	5.17 ± 0.06 ^yc^	5.18 ± 0.07 ^c^	5.16 ± 0.04 ^c^
	Superchilling (−4 °C)	6.06 ± 0.12 ^a^	6.27 ± 0.19 ^xa^	5.62 ± 0.02 ^xb^	5.36 ± 0.06 ^xc^	5.04 ± 0.09 ^d^	5.13 ± 0.07 ^d^
ATP (μmol/g)	Chilling (4 °C)	45.07 ± 1.63 ^a^	43.09 ± 2.11 ^a^	41.83 ± 1.55 ^xyab^	37.54 ± 1.74 ^yc^	33.60 ± 1.03 ^yd^	32.31 ± 1.66 ^d^
	Supercooling (−1 °C)	44.87 ± 1.51 ^a^	42.65 ± 0.16 ^ab^	40.35 ± 2.06 ^ybc^	36.51 ± 2.58 ^yc^	33.48 ± 1.98 ^ycd^	31.54 ± 1.66 ^d^
	Superchilling (−4 °C)	45.62 ± 0.99 ^a^	44.88 ± 2.24 ^a^	43.91 ± 0.58 ^xa^	43.08 ± 0.32 ^xa^	37.57 ± 1.12 ^xb^	31.22 ± 1.12 ^c^
Glycogen (mg/g)	Chilling (4 °C)	3.44 ± 0.06 ^a^	2.85 ± 0.11 ^yb^	2.17 ± 0.06 ^yc^	1.76 ± 0.12 ^yd^	1.39 ± 0.04 ^ye^	0.99 ± 0.06 ^yf^
	Supercooling (−1 °C)	3.47 ± 0.05 ^a^	3.06 ± 0.01 ^xb^	2.43 ± 0.12 ^xc^	2.13 ± 0.11 ^xd^	1.88 ± 0.08 ^xe^	1.28 ± 0.01 ^xf^
	Superchilling (−4 °C)	3.47 ± 0.03 ^a^	2.65 ± 0.12 ^yb^	1.95 ± 0.03 ^zc^	1.36 ± 0.11 ^zd^	1.10 ± 0.02 ^ze^	0.85 ± 0.04 ^zf^
Lactate (mmol/g)	Chilling (4 °C)	49.82 ± 0.30 ^c^	51.63 ± 0.94 ^c^	53.77 ± 0.55 ^zb^	56.14 ± 0.54 ^ya^	57.69 ± 0.63 ^ya^	60.06 ± 0.88 ^a^
	Supercooling (−1 °C)	49.84 ± 1.13 ^e^	51.67 ± 0.71 ^d^	55.35 ± 1.12 ^ycd^	57.08 ± 0.29 ^ybc^	58.68 ± 0.17 ^yab^	61.21 ± 0.42 ^a^
	Superchilling (−4 °C)	49.80 ± 0.47 ^d^	51.53 ± 0.46 ^c^	59.34 ± 0.25 ^xb^	59.05 ± 0.46 ^xb^	63.88 ± 0.59 ^xa^	59.98 ± 0.47 ^b^

The results were shown as means and standard errors. x–z: letters denote statistically significant inter-group differences at the same time point (*p* < 0.05). a–f: letters indicate significant intra-group differences over different storage durations (*p* < 0.05).

## Data Availability

The original contributions presented in the study are included in the article, further inquiries can be directed to the corresponding authors.

## Abbreviations

The following abbreviations are used in this manuscript:

VFC	very fast chilling
WHC	water-holding capacity
PYGM	glycogen phosphorylase
PFKM	phosphofructokinase
PGK	phosphoglycerate kinase
ALDOA	aldolase
LDH	lactate dehydrogenase
TPI1	triosephosphate isomerase
PVDF	polyvinylidene fluoride membrane
NC	nitrocellulose membrane
GLM	general linear model
